# Satellite Glial Cells in Pain Research: A Targeted Viewpoint of Potential and Future Directions

**DOI:** 10.3389/fpain.2021.646068

**Published:** 2021-03-10

**Authors:** Parisa Gazerani

**Affiliations:** ^1^Laboratory of Molecular Pharmacology, Department of Health Science and Technology, Faculty of Medicine, Aalborg University, Aalborg, Denmark; ^2^Pharmacy, Department of Life Sciences and Health, Faculty of Health Sciences, OsloMet, Oslo, Norway

**Keywords:** satellite glial cells (SGCs), pain, sensory ganglia, trigeminal ganglion (TG), dorsal root ganglion (DRG), nociception, peripheral nervous system

## Abstract

Chronic pain is known to be caused by sensitization within the pain circuits. An imbalance occurs between excitatory and inhibitory transmission that enables this sensitization to form. In addition to neurons, the contribution of central glia, especially astrocytes and microglia, to the pathogenesis of pain induction and maintenance has been identified. This has led to the targeting of astrogliosis and microgliosis to restore the normal functions of astrocytes and microglia to help reverse chronic pain. Gliosis is broadly defined as a reactive response of glial cells in response to insults to the central nervous system (CNS). The role of glia in the peripheral nervous system (PNS) has been less investigated. Accumulating evidence, however, points to the contribution of satellite glial cells (SGCs) to chronic pain. Hence, understanding the potential role of these cells and their interaction with sensory neurons has become important for identifying the mechanisms underlying pain signaling. This would, in turn, provide future therapeutic options to target pain. Here, a viewpoint will be presented regarding potential future directions in pain research, with a focus on SGCs to trigger further research. Promising avenues and new directions include the potential use of cell lines, cell live imaging, computational analysis, 3D tissue prints and new markers, investigation of glia–glia and macrophage–glia interactions, the time course of glial activation under acute and chronic pathological pain compared with spontaneous pain, pharmacological and non-pharmacological responses of glia, and potential restoration of normal function of glia considering sex-related differences.

## Introduction

Chronic pain is a debilitating and common condition (1), and it has a substantial impact on affected individuals, society, and the health-care system (2). It is generally accepted that pathological chronic pain is caused by a maladaptive process that occurs when an imbalance is present between excitation and inhibition signaling pathways underlying pain (3). Both functional and structural alterations have been identified. Altered neuronal activity, manifested as sensitization of peripheral primary sensory neurons in the sensory ganglia [e.g., dorsal root ganglia (DRG) and trigeminal ganglia (TG)] and central sensitization of nociceptive neurons within the central nervous system (CNS), including the spinal cord, trigeminal nucleus, brain stem, and cortex, has been reported (4). Treatment of chronic pain is complicated and often results in inadequate response or side effects. Attempts are ongoing for a better understanding of pain processes, mechanism-based treatment and targeting, implications of multidisciplinary pain management, and patient-centered strategies (5).

Generally, there has been increasing interest in the role of the non-neuronal components of the nervous system (glial cells) in the health and diseases of the nervous system (6–8). These cells have been markedly recognized to contribute to the development or maintenance of abnormal neuronal excitability (9). In this line, accumulating evidence supports the contribution of glial cells in the initiation or maintenance of chronic pain (10). Major glial residents in the CNS, namely, astrocytes and microglia, have been the subject of extensive research, and their important role in the pathogenesis of persistent pain is becoming definitive (11–13). Cross talk between astrocytes, microglia, and neurons has been suggested to promote pathological chronic pain or pain chronification, i.e., transition from acute to chronic pain (14). Interestingly, in the context of pain, gliopathy (e.g., astrogliosis and microgliosis) seems to play distinct roles (10, 15). Gliosis is non-specifically defined as a reactive response of glial cells in response to insults to the CNS. Differences in the response of microglia and astrocytes depend on the type of pain (16), the time course of insult (17), and sex (18). Excellent reviews are available to comprehend the role of astrocytes and microglia in chronic pain (10, 19–24). Recently, the potential role of other central glia, oligodendrocytes, has also been investigated, and current findings collectively support their participation in the central pain process and contribution to persistent pain (25). Targeting central glia to reverse chronic pain or to prevent its development has also emerged (26, 27).

Glial cells of the peripheral nervous system (PNS) have also been investigated in the context of chronic pain pathology and targeting (28–30). These cells include satellite glial cells (SGCs), Schwann cells (SCs), and enteric glial cells (EGCs). The latter two cell types are less investigated than SGCs. Within ganglia, SGCs surround the cell bodies of neurons very closely and create a unique structure, a unit of neuron–SGC, which is not found in other parts of the nervous system (31). In different pain models with a neuropathic or inflammatory nature, SGCs have been shown to undergo alterations in structure and function (31, 32). Consequently, the neuronal activity of sensory ganglia neurons is affected, which is reflected in hyperactivity of neurons, neuron–SGC coupling, elevated responses to adenosine triphosphate (ATP), release of cytokines, and downregulation of potassium channels (32). It is proposed that this increase in neuronal activity is linked to the development of chronic pain. A distinct pattern is seen in SGCs following insult to the PNS. A recent review summarized common changes that occurred in SGCs in four major pain models: systemic inflammation, postoperative pain, diabetic neuropathy, and postherpetic pain (32). SGC alterations have been documented in response to both injury and inflammation. These cells, therefore, have become another potential target for therapeutic purposes, i.e., for the prevention or treatment of chronic pain. An argument has been formed around preference in targeting these cells, as SGCs are located outside the blood–brain barrier (BBB), which might offer a better potential for blocking pain transmission at the periphery. Considering that these cells seem first to respond to injury or inflammation prior to central glial cells, they may also offer potential for minimizing the risk of chronification and transition from peripheral to central sensitization (15, 33). Elegant reviews are available to deepen the knowledge of what has been investigated and found in exploring the roles of SGCs in pain (32, 34–37) or its targeting (27). The purpose is therefore not to provide a comprehensive systematic review of the current literature on the role of glial cells in pain, since several excellent reviews are already available, where the readers are referred to (10, 19–24, 32, 34–37). Instead, this paper aims to provide a viewpoint on potential future directions and avenues to stimulate further interest and to form scientific hypotheses with a focus on peripheral glia, mainly SGCs. Further investigation of glia in relation to pain and its targeting is not only a truly fascinating field of science but also highly valuable in understanding pain mechanisms and mechanistic-based optimized targeting.

## Future Directions For Satellite Glial Cells in Pain Research

### Satellite Glial Cells' Characterizations by Aid of Novel Tools

Historically, SGCs were considered cells that share some common features with astrocytes; hence, the expression of some proteins that were known for astrocytes was expected in these cells, such as glial fibrillary acidic protein (GFAP), glutamine synthetase, glutamate aspartate transporter, and connexin 43 gap junction (31, 38). However, it was determined that these cells have their own morphology and characteristics that are unique, and differences might exist between SGCs that are located in the DRG and those located in the TG. Heterogeneity was also observed in terms of the morphology and distribution of these cells within sensory ganglia around different neuronal populations, e.g., with different sizes (39). These observations highlighted the fact that the characterization of these cells in the TG and DRG under physiological and pathological pain with different natures (e.g., neuropathic and inflammatory) is valuable. This is crucial when pain conditions in humans are modeled in laboratory animals and to test potential targets for pain. It has gradually become evident that accurate information on SGC and macrophage morphology and function will facilitate research on the roles of these cells in pain and as potential therapeutic targets (15, 28). Perhaps one of the limitations that have slowed down the process has been the lack of proper methods or tools to facilitate dynamic visualization of these cells.

A recent study (40) focused on the characterization of SGCs and macrophages in the DRG. The authors applied the method of specific gene expression or deletion and examined Ca^2+^ dynamics in these cells. Both immunohistochemistry and 2-photon Ca^2+^ imaging have been used to characterize SGCs in the DRG in the available and most commonly used genetically modified mouse lines that are used to study astrocytes or microglia. Interestingly, findings from this study pointed out that the majority of lines used in studying astrocyte functions were not efficient in studying SGCs in the DRG, with the exception of two mouse lines. The authors used mouse lines of S100β-eGFP, ALDH1L1-eGFP, GFAP-Cre::GCaMP6f, GLAST-CreERT2::GCaMP6f, Cx30-CreERT2::GCaMP6f, and Cx43-CreERT2::GCaMP6f for SGCs in the DRG and similar lines for astrocytes in the visual cortex (40). The double transgenic line Cx43-CreERT2::GCaMP6f permitted inducible GCaMP6f expression in more than 90% of DRG SGCs (92.6%), where the expression of GCaMP6f in neurons was only 4%. It remains to be determined whether GCaMP6f is expressed in other cells within the DRG, such as endothelial cells, fibroblasts, or SCs (40). Interestingly, not only was the expression of Cx43 found to be very stable, but also it was upregulated after injury insult in the PNS (40). Hence, it seems that this mouse line can be a useful tool in pain research focused on PNS and pain. The results from this study (40) also demonstrated that the knock-in CX3CR1-eGFP mouse line presents specific eGFP expression in the majority of microglial cells and macrophages in both the DRG and the visual cortex. Therefore, this line can be an option when studying specific targeting of SGCs in the DRG. These two validated mouse lines (Cx43-CreERT2::GCaMP6f and CX3CR1-eGFP) could be used as proper tools for further investigation of SGCs in the DRG under healthy and painful conditions (40). This direction presents new avenues toward the development and application of research tools to enable progress in research on SGCs in relation to pain. For example, it has been proposed that genetically encoded animals can allow studying sensory neuron–SGC interactions (41, 42). This would provide potential for studying the specific roles of target genes that are expressed in SGCs following pathological pain. This approach has similarly been proposed for investigating the roles of SCs in neuropathic pain (30).

Another attempt is to properly isolate SGCs to characterize them and study their function (43). This approach has been used to examine whether isolation would dramatically change the natural milieu that SGCs normally experience *in vivo*. A contradiction exists in the literature, but cell-based platforms have been used for the characterization of SGCs (44–47) and their function (48, 49). Recent studies have shown that the transcriptomes of SGCs can be determined under normal and pain conditions (50). Next-generation RNA sequencing by Jager et al. (50) provided the first evidence on the state of SGCs under normal conditions and following peripheral nerve injury. Findings from this study show similarities between naïve SGCs and astrocytes, being enriched in genes associated with the immune system and cell-to-cell communication. Data from this study (50) show that 3 days following injury, several genes linked to cholesterol biosynthesis are downregulated in SGCs, and this pattern was also present 14 days postinjury. SGC transcriptional analysis, however, shows a signature that 14 days postinjury, a higher expression of genes associated with MHCII and migration of leukocytes is present. Access to the full transcriptome has been offered by the authors (on the gene omnibus database) (50) and can serve as an important and valuable tool to understand cell function and regulation of different gene products. This study is also the first to provide evidence that postinjury perineuronal proliferating cells are not SGCs but macrophages.

Transcriptomics, focused on the characterization of individual cells, is increasingly used (51). This approach is valuable because single-cell RNA sequencing allows analysis of subtypes of SGCs and comparison of these cells in different sensory ganglia in one species or comparison between species, for example, between rodents and humans. This line of research will be particularly important when researchers are focused on pain conditions that are specific to one type of sensory ganglia, for example, dental pain, headache, and other types of orofacial pain that need a focus on SGCs in the TG (52, 53).

In addition, due to the nature of translational gaps between human glia and glia in rodents (54), the identification of human sensory ganglion cells would reveal similarities and differences and hence provide a more accurate understanding based on transcriptome profiling. Some attempts have already been initiated (55, 56). Having access to human sensory ganglia (healthy and pain patients) for research would close the gaps in the findings obtained from rodent models (32). A study in 2018 (55) examined the transcriptomic analyses of DRGs obtained from human donors and mouse tissues, including DRGs. This study has also created an online, searchable repository to provide access to data on cross-species analysis of DRGs. This would be highly valuable to speed up the screening of valuable targets for therapeutic purposes (55). This indeed also emphasizes an urgent need to access databases for researchers working in SGC-related pain research.

Extracellular vesicles (EVs) are released by cells into the extracellular space. EVs are secreted by a range of cell types, can be isolated, and can be characterized. Their roles in the nervous system (e.g., in cell–cell communication) under health and disease have been reviewed recently (57). Proteomic profiling of EVs shed from SGCs has been reported (45), and the findings have revealed differentially regulated proteins when SGCs are stimulated by lipopolysaccharides (LPSs; mimicking inflammation). These proteins include junction plakoglobin and myosin 9, which can be considered markers of SGC responses under inflammatory conditions.

An elegant recent review (58) summarized the ncRNAs in neuropathic pain within the PNS and the CNS. The findings cover both neuronal and non-neuronal cell sources of these molecules and, interestingly, those related to SGCs in the TG and DRG. For example, NONRATT021972 and uc.48+ upregulate the ionotropic purinoreceptor P2X7 in SGCs (59, 60). Interestingly, inhibition of uc.48+ has been shown to reduce mechanical hypersensitivity in a rat model of trigeminal neuralgia by inhibiting the expression of the P2X7 receptor in trigeminal SGCs (61).

ncRNAs' roles in pain are not limited to neuropathic pain. A recent study (62) provided information on the role of lncRNA X inactivate-specific transcript (XIST) in inflammatory pain. In this study, a complete Freund's adjuvant (CFA) model of inflammatory pain was established in rats, where high expression of XIST and voltage-gated sodium channel (VGSC) 1.7 (Nav1.7) was observed in the DRG. When the authors applied XIST inhibition, pain behavior (reflected on mechanical withdrawal threshold) and SGC expression of GFAP, inflammatory cytokine levels of interleukin-6, and tumor necrosis factor-α were diminished (62). In contrast, downregulation of XIST increased the mechanical pain threshold and decreased the expression of miR-146a. To identify the role of XIST, the authors ran an *in vitro* test and identified that XIST acted as a sponge of miR-146a, which targeted Nav1.7 and concluded that based on these observations, XIST can regulate SGCs in the DRG under inflammatory pain condition and hence can be a future therapeutic target (62).

Therefore, the identification of signatures or biomarkers in SGCs can offer a further characterization of these cells under health and pathological pain conditions. The literature presents some data available for both the DRG (50) and TG (45, 63). lncRNAs and circRNAs and the computational construction of interaction networks between lncRNAs/circRNAs–miRNAs–mRNAs can provide new directions and potential therapeutic targets.

### Computational Modeling of Satellite Glial Cells' Behavior Within the Sensory Ganglia

Another path that researchers started exploring is the potential of computational modeling. For example, a group of researchers (64) have tried to investigate and determine the characteristics of intercellular communication between sensory neurons in the DRG and SGCs by applying ATP. Researchers of this study have proposed that the neural engineering approach provides a physiologically constrained computational model that can be used for several purposes, in addition to physiological communication of neurons and SGCs (64), for example, understanding of various factors that control this communication, such as changes in receptor expression or activity, e.g., Kir 4.1 current density that occurs in SGCs under pain. Perhaps by expansion in the use of artificial intelligence in neural engineering, this field can also benefit from further advancement to deepen the knowledge on predictive parameters affecting SGC–neuron interactions in relation to pain. Such an attempt has been presented for neuron–astrocyte interactions (65). Biocomputational modeling can potentially provide a platform to test hypotheses about SGC–neuron interactions or SGC–SGC interactions and parameters influencing those interactions within the sensory ganglia.

### Satellite Glial Cells' Role in Nerve Repair

Research on nerve repair has long focused on sensory neurons and their signaling alterations after injury in addition to SCs that insulate axons (66–68). Only recently have sparks been raised about the contribution of SGCs that envelop the neuronal soma. Evidence started to accumulate supporting their roles in nerve repair. A recent study (69) provided results indicating that the synthesis of fatty acids in SGCs promotes sensory neuron repair after injury and results in regeneration. In this study (69), first, the researchers identified a new marker in SGCs via transcriptional profiling, which is called Fabp7/BLBP (fatty acid binding protein 7/brain lipid-binding protein). Upon nerve injury, alterations in gene expression were observed in SGCs that were mainly related to fatty acid synthesis and peroxisome proliferator-activated receptor alpha (PPARα) signaling. Based on this observation, researchers (69) modeled the injury condition, where deletion of fatty acid synthase (Fasn) resulted in the absence of axon regeneration. To reverse this condition, they applied fenofibrate, which is a PPARα agonist, and axon regeneration returned in mice lacking Fasn in SGC. These findings (69) demonstrated that fatty acid synthesis in SGC is a crucial step in nerve repair in adults after peripheral nerve injury. In the context of pain, this can offer a new direction in regenerative responses after nerve injury promoted by SGCs. Interestingly, astrocytes have been identified as essential for the development and function of axons *in vivo*, and lipid metabolism in these cells has been found to be a critical step in this process (70). Therefore, the authors of this study (69) have suggested further investigations to identify how lipid metabolism in SGCs influences axon regeneration, for example, via a paracrine effect or other mechanisms. In addition, they left open questions for further investigation of the potential effects of fenofibrate on centrally projecting sensory axon growth (69). The clinical implication of fenofibrate to yield beneficial neuroprotective effects has already been discussed for diabetic retinopathy (71) and brain trauma (69, 72). Considering the complexity of the changes that occur after peripheral nerve injury and the involvement of several cell types, it has been suggested to investigate interactions between SCs, fibroblasts, and macrophages in addition to sensory nerves and SGCs (73). This would facilitate a better understanding of cell-specific roles in repair phenomena following peripheral nerve injury. In addition, much remains to be investigated in relation to myelinating and non-myelinating forms of SCs (74). Identification of cell–cell interactions might be achievable by new high-resolution live imaging techniques (75) to characterize dynamic changes in neuropathies over time, e.g., changes in SGCs of damaged nerves or development of new SGCs, and identification of acute vs. chronic responses for event time-course analyses. In addition, it has been demonstrated that macrophages interact with SGCs within sensory ganglia (76). Therefore, the interaction of SGCs with other cells is valuable to consider in future studies and how the interaction may influence the overall neuronal response.

It has been shown that transplantation of SCs might be a promising method to promote neural repair. SCs from rats were cultured and microencapsulated in a research study (77) and then administered to rats that underwent chronic constriction injury (CCI). Data showed that microencapsulated SC transplantation could block the expression of the purinergic receptor P2X3 in the DRG and diminish the behavioral components of a neuropathic pain model (77). It is not yet known whether such a method can be applicable for SGCs considering that fatty acid synthesis in SGCs has been identified as a crucial step in nerve repair in adults after peripheral nerve injury (69).

An increased number of studies are becoming available to present the responses of SCs, in particular to nerve injury and contributions to neuropathic pain (30, 78). An emerging line of investigation related to SCs in pain is the identification and characterization of different roles of myelinating and non-myelinating SCs in neuropathic pain. There is also interest in drugs that can target SCs in addition to the possibility of SC transplantation as potential future options in the treatment of neuropathic pain (30).

Recently, a specialized type of peripheral glial cell was discovered in the skin (79), where it produces a mesh-like network that plays an essential role in sensing noxious stimuli to thermal and mechanical stimuli. These glial cells are closely associated with unmyelinated nociceptors and convey nociceptive information to the nerve; hence, they are called nociceptive SCs. Further investigation is expected to emerge on these cutaneous SCs and their role, now that they have been found to be able to initiate pain-like behavior (79).

### Functional Roles of Satellite Glial Cells

A general view is that activation of glial cells contributes to the development of pain due to the release of proinflammatory cytokines and chemokines and other substances and factors that drive pain signaling, such as glutamate, calcitonin gene-related peptide, and substance P (80). However, since glial cells also release anti-inflammatory substances, one can consider that beneficial effects might also be present, for instance, to reverse neurotoxicity and pain (80). Considering this side of the coin, we might be able to promote the protective function. This is particularly interesting, as glial inhibitors *per se* have not been successful in alleviating pain, mainly because the normal activity of glia must remain reserved, as they have critical roles with the PNS and CNS. This is not an easy path in the production of glia-associated drugs because the way that glial cells behave is complex and depends on numerous factors, such as the type of stimuli, location, and length of stimuli. Information on SGCs is very limited in this area, but some literature exists for microglia and astrocytes. The challenge is still to determine whether and how the proinflammatory nature of SGC activation can be prevented while its anti-inflammatory nature can be promoted. It has been shown that activation of central glia by LPS leads to the release of proinflammatory cytokines, but when growth factors or anti-inflammatory cytokines are applied, glial cells release factors that can promote neuronal survival (80).

This field needs further *in vitro* research (e.g., rodent and human cell cultures), *in vivo* research (e.g., transplantation of human glia in rodents), and translational research in humans [e.g., by application of positron emission tomography (PET) technique and tracers (81, 82), to follow glial activation at different time points and in response to different stimuli or in pain patients with acute or chronic pain conditions]. Eventually, by better understanding the molecular mechanisms behind the role of glia in pain, proper, and safer therapeutic agents might be developed. Focusing on central glia in this line does not necessarily close the field for more research in peripheral glia, including SGCs. In addition, considering different pain conditions, one can reserve possibilities for the activation of SGCs in the TG that contributes to orofacial and craniofacial pain conditions vs. their activation in the DRG that relates to pain in other body regions, even though overlap occurs, for example, in diabetic neuropathy manifested in the feet and eye (83) or musculoskeletal pain (84).

### Sex-Dependent Characteristics and Function of Satellite Glial Cells

Considering that pain is a sexually dimorphic phenomenon (85) and that some painful conditions are predominant in one sex (e.g., migraine in females) or only exist in one sex (e.g., pelvic pain due to endometriosis in females), it is important to include this aspect in further glial-associated pain research (86). A number of reports propose that pathological pain in males is regulated by microglial signaling (21, 87); however, astrocyte signaling seems not to show a sex-dependent nature in inflammatory and neuropathic pain models (18).

The literature shows that following peripheral nerve injury, proliferation, and morphological changes occur in microglia in males and females (85). However, only in male animals has the functional role of microglia been observed, which is proposed to drive neuropathic pain (88, 89).

We still do not know whether any sex-related characteristics or functional responses exist in the activation of SGCs following PNS insult. Further research can present the value and importance and whether any natural protective mechanism or susceptibility might exist in either sex related to SGCs and whether this can be manipulated or targeted for pain control.

### Satellite Glial Cells in Sympathetic and Parasympathetic Ganglia

The behavior of SGCs in the sympathetic ganglia has rarely been investigated (32, 90); hence, the role of these cells is not yet clear. A study from 2004 (91) reported that sciatic nerve transection resulted in changes in both the DRG and lumbar sympathetic ganglia, where neuroinflammatory responses were evident in both ganglia, and interestingly, some markers were more affected in the sympathetic ganglia than in the DRG, such as GFAP reactivity, macrophage reactivity, and T cell responses (91).

Another study examined the recruitment of T-lymphocytes and macrophages into lumbar sympathetic ganglia and DRG in a rat model of spinal nerve ligation (SNL), where different patterns of response were found. The authors suggested that this pattern difference between these ganglia may provide information about contribution of macrophages in neuronal insult and post injury hyperexcitability (92). Another study (93) demonstrated that when the sympathetic nerves in the superior cervical ganglia were damaged, SGCs became activated and underwent alterations consisting of coupling, higher sensitivity to ATP, and less responsiveness to acetylcholine. Interestingly, in this study, SGCs of the TG were not affected (93).

Glial coupling is not limited to autonomic ganglia and has also been studied in sensory ganglia (41, 94). Coupling is defined as the formation of connectivity between SGCs that can be observed as an elevated number of gap junctions between these cells, which was reported in response to peripheral nerve injury (37). Next, electrophysiological methods and dye injection were applied to confirm the initial observations that higher coupling occurred postinjury. This finding has been reported consistently in the literature in several pain studies in which both inflammatory and neuropathic models were applied (95, 96). Consequently, it was reported that gap junction blockers such as carbenoxolone, meclofenamic acid, and palmitoleic acid could diminish coupling between SGCs and reduce pain in experimental animal models (97). Collectively, evidence supports the notion that enhancement of SGC coupling through gap junctions is associated with the development and maintenance of neuropathic pain (37).

Among connexins, connexin 43 (Cx43), a gap-junction protein that is expressed in activated SGCs (98), has been investigated rather extensively. This has gained attention because connexins could be targeted to block pain. Connexin proteins are presented with 20 subtypes and, among other roles, function as gap junctions between cells. Recent studies highlight the role of connexins in the induction and maintenance of chronic pain, where their modulation has resulted in pain amelioration in several chronic pain models (99). Interestingly, chemotherapy-induced neuropathic pain, for example, following the administration of oxaliplatin and taxol, has been linked to SGC activation, with a proposed mechanism involving coupling by gap junctions (100). This has also been shown *in vitro* (101). Blocking gap junctions, for example, by administration of carbenoxolone, which blocks connexin 43, has been shown to reduce chemotherapeutic-induced hypersensitivity in animal models of pain (100).

Collectively, and based on limited available data (102), research on SGC activation, coupling, and their influence on neurons within autonomic ganglia (sympathetic and parasympathetic) and sensory ganglia—in relation to pain—will continue to emerge (32, 103). Further investigation would also help identify the exact underlying mechanisms of gap junctions and inhibitors in pain control (90). Drug-induced peripheral neuropathy, as has been seen with chemotherapeutic agents, can also be studied further, and research should examine the role of SGCs in limiting the side effects of these agents.

### Miscellaneous

In addition to pharmacological approaches as powerful tools to study SGCs in pain research, it is also of great interest to test non-pharmacological approaches, for example, whether neuromodulation techniques that are used to alleviate pain of different types could exert any effect on non-neuronal cells in chronic pain.

Investigation of the effects of environmental factors on SGCs, such as dietary control, microbiome, and other environmental factors, such as stress, under health and pathological pain conditions remains open.

The role of the SGC in the overall immune responses in ganglia, for instance, pathogen defense against viral infection, is becoming more evident. This avenue might not be directly linked to pain research but will allow for further characterization of the non-neuronal response of SGCs within PNS ganglia.

EGCs reside within the enteric nervous system (ENS) (104). These cells have some common features with astrocytes from the functional and structural points of view. EGCs regulate ENS homeostasis, and violation of their normal function leads to gastrointestinal disorders, such as functional gastrointestinal disorders and inflammatory bowel diseases (105, 106). In addition, EGCs have been identified to modify visceral pain signaling via cross talk with neurons and immune cells. This observation has potential in understanding the mechanisms underlying chronic abdominal pain and its targeting (107, 108). This direction of research is also proposing to expand further, in particular in relation to an increasing amount of research on gut microbiota and its interaction with the brain. In addition, since enteric glia are affected by stress, they are considered to play a substantial role in visceral hypersensitivity and the immune response to stress (107).

## Conclusion

In the past few years, several breakthroughs have been achieved with a focus on glia and cross talk of glia with neurons and other cells that have revolutionized pain research and inspired implications for pain management in the future and further research.

The development and availability of sophisticated technology and tools (109) to study the molecular, genetic, morphological, physiological, and pathological aspects of glia *in vitro* and *in vivo* have definitely advanced the field. Translational research has moved the field from experimental rodent models toward human studies, although limited, but substantial achievements have been made. Some clinical trials have attempted to use available compounds with glial modulatory effects in humans with various outcomes, such as vitamin D (110) and ibudilast (111) in migraine patients, minocycline for lumbar radicular neuropathic pain (112), and palmitoylethanolamide for the treatment of different types of pain (113).

Investigation of human glia moves the field one step closer to testing and potential application of strategies for prevention and therapy of chronic pain, with fewer risks due to interspecies gaps from preclinical to clinical stage. Realization of parameters that can influence the complex behavior of glia has also advanced formulation of testable hypotheses, for example, interactions of SGCs with other SGCs, neurons, and macrophages that collectively determine pain responses to nerve injury and inflammation. Considering age and sex is gaining further attention in studying glia in pain research.

Bioinformatics, neuronal engineering, complex modeling, and dynamic and live assessment techniques together with profiling these cells via quantitative methods such as mapping the transcriptome and evaluating the responses of SGCs to indirect environmental changes in the host that can influence pain response and sensitivity have become more familiar to researchers and have inspired drug designer and pharmaceutical and non-pharmaceutical strategies to maintain the protective and positive functions of SGCs while shifting their negative influence on pain toward pain relief.

In addition, finding the crucial role of SGCs in nerve repair deserves further investigation. A focus on peripheral nerve regeneration via the contribution of both SGCs and SCs sounds logical. At a system level, one can also consider how much knowledge can be obtained if information can be collected from different types of glia within different systems, e.g., from both PNS and CNS glia, considering the time course of acute and chronic pain and response to manipulating factors. This would enhance the visibility of the big picture in the entire body system to unmask some missing pieces.

These and several more dimensions, such as 3D tissue prints, potential use of induced pluripotent stem cells (iPS cells), and cell transplantation techniques, with a wide range of research applications in this field, have become available and should be further researched to not only advance the fascinating science of glia but also to pave the way for potential targeted therapy that can offer safer and efficient options for pain patients.

## Author Contributions

PG conceptualized and carried out the literature search and wrote this mini review.

## Conflict of Interest

The author declares that the research was conducted in the absence of any commercial or financial relationships that could be construed as a potential conflict of interest.
